# Prognostic value of EMT-related genes and immune cell infiltration in thyroid carcinoma

**DOI:** 10.3389/fimmu.2024.1463258

**Published:** 2024-11-04

**Authors:** Shuping Wu, Yu Liu, Yu Zeng, Xianhui Ruan, Mei Tao, Wenrong Lin, Chang Liu, Hongbin Chen, Hui Liu, Yu Wu

**Affiliations:** ^1^ Department of Head and Neck Surgery, Clinical Oncology School of Fujian Medical University, Fujian Cancer Hospital, Fuzhou, Fujian, China; ^2^ Department of Thyroid and Neck Tumor, Tianjin Medical University Cancer Institute and Hospital, National Clinical Research Center for Cancer, Tianjin’s Clinical Research Center for Cancer, Key Laboratory of Cancer Prevention and Therapy, Tianjin, China; ^3^ Department of Ultrasound, Clinical Oncology School of Fujian Medical University, Fujian Cancer Hospital, Fuzhou, Fujian, China

**Keywords:** THCA, EMT, CTSK, immune infiltration, biomarker

## Abstract

**Background:**

The Epithelial–Mesenchymal Transition (EMT) is a very important process involved in cancer invasion and metastasis. Additionally, the Cathepsin K (CTSK) gene is closely related to the degradation of the extracellular matrix, which is a critical component of the EMT. The purpose of this study was to determine the relationships between EMT-related genes and immune cell infiltration and their prognostic value in Thyroid carcinoma (THCA). The effect of the CTSK gene on the aggressive biological features of THCA was assessed.

**Methods:**

Within the framework of the present study, the THCA cohort was analyzed in detail based on data obtained from The TCGA database in the context of the EMT. The TCGA-THCA cohort was then divided into two groups, namely, high- and low-risk groups, based on the calculated EMT scores. Finally, based on the findings from the Weighted Gene Co-Expression Network Analysis (WGCNA) algorithm, LASSO regression analysis, and Kaplan−Meier plotter, we selected five genes (CTSK, C3ORF80, FBLN2, PRELP and SRPX2) associated with patient prognosis. Furthermore, this study examined the presence of various immune cells within the THCA samples using three distinct algorithms, namely ssGSEA, xCell, and MCPcounter. Additional studies have been conducted to establish the roles of CTSK in THCA cell proliferation and migration using various assays, such as CCK8, colony formation, EdU proliferation, Transwell migration and wound healing assays. Additionally, the involvement of CTSK in the regulation of various EMT-related markers was confirmed using Western blot analysis.

**Results:**

Based on EMT scores, TCGA-THCA patients were further divided into two groups, and the study revealed that patients in the high-risk group had a worse prognosis than those in the low-risk group. Among the five genes linked to the prognostic value of EMT (CTSK, C3ORF80, FBLN2, PRELP, and SRPX2), CTSK exhibited notably elevated expression in the high-risk cohort. This group also exhibited pronounced immune cell infiltration, with a marked correlation observed between CTSK expression and the levels of macrophages, MDSCs, and various T-cell subtypes. Furthermore, *in vitro* studies demonstrated that reducing CTSK expression led to significant reductions in THCA cell viability; clonogenic, proliferative, motility and migratory capacities; and the expression of key EMT-related proteins, including N-cadherin, vimentin, slug, and snail.

**Conclusion:**

Our results suggest that the expression of CTSK, a gene associated with the EMT, may be associated with THCA onset and progression and thus may serve as a promising prognostic biomarker.

## 1 Introduction

Thyroid cancer (THCA), which originates from either follicular or parafollicular epithelial cells within the thyroid gland, is the predominant malignant endocrine tumor, accounting for approximately 90% of all malignancies within this system (1–3). Despite the generally favorable prognostic outlook for most cases of THCA, specific forms, such as anaplastic, medullary, and treatment-resistant thyroid cancers, demonstrate a propensity for recurrence and metastasis, ultimately leading to detrimental outcomes (4, 5). In response to these challenges, recent therapeutic advancements have included the adoption of sorafenib, a multitargeted small-molecule tyrosine kinase inhibitor that acts on VEGFR, BRAF, and RET. Although beneficial for managing advanced or metastatic forms of THCA, the application of sorafenib is frequently hampered by its potential to trigger a spectrum of adverse effects within patients (6). The limitations of current treatment options emphasize the pressing necessity of identifying and validating novel genetic markers. These markers not only gauge the aggressive nature of tumors but also facilitate the development of targeted treatments, thereby enhancing management strategies for patients suffering from THCA with an otherwise poor prognosis.

The epithelial–mesenchymal transition (EMT) is a process in which epithelial cells transform into mesenchymal cells, as described previously (7). This process is indispensable for embryonic development and wound healing and has a major impact on tumor growth and metastasis. This dual role of EMT in normal wound healing and pathophysiological processes such as cancer portrays the significance of EMT in living organisms. These interactions include promoting tumor cell motility and invasion, increasing tumor cell stemness, and increasing tumor cell chemoresistance and immunoresistance. The process known as EMT is intricately governed by an extensive range of factors that originate both internally within cells and externally from the cellular environment. These regulatory elements include numerous transcription factors, diverse mechanisms of posttranslational modification, comprehensive epigenetic changes, and various noncoding RNAs (8). Various studies have established that the EMT does not operate as a straightforward binary mechanism. In contrast, this process occurs in a step-by-step manner through several well-coordinated cellular stages (9). The connection between EMT and MSCs in THCA is crucial, and their interaction might be responsible for the poor outcome of patients with certain THCA subtypes through the stimulation of MSC-like cell proliferation for metastasis (10). Some papillary tumors are associated with metastatic and invasive behaviors despite the fact that most of the thyroid tumors are well differentiated because of dedifferentiation. This finding can be explained by the EMT, whereby thyroid epithelial cells undergo a transition, assume a fibroblastic morphology, become less cohesive and more motile and express mesenchymal markers (11). The tumor microenvironment (TME) has been described as a complex structure composed of both living and nonliving components. Other cell types that constitute this environment include endothelial cells, adipocytes, fibroblasts, epithelial cells and immune cells in addition to primary tumor cells. Moreover, the TME includes acellular components, including the extracellular matrix (ECM), cytokines, chemokines, growth factors and antibodies, which are involved in carcinogenesis and tumor advancement (12, 13). Several works have emphasized a strong correlation between a high level of immune cell infiltration in tumor tissue and patient survival. The relationships between the elements of an individual’s antitumor defense and the features of the tumor, including the rates of tumor growth, invasion, and metastasis, influence the response to therapy and the predicted course of THCA (14). Cathepsin K (CTSK) is a ubiquitously expressed protease that plays enzymatic and nonenzymatic roles in numerous pathologies (15). Several recent studies have shown a strong correlation between increased CTSK levels and the onset and poor prognosis of pancreatic and hepatocellular carcinomas. Additionally, higher CTSK levels promote disease progression to the lymph nodes in patients with oral squamous cell carcinoma (16–18). It should be noted that CTSK is reportedly involved in the promotion of an M2-like macrophage phenotype in castration-resistant prostate cancer (19). However, it remains unclear how CTSK levels are associated with THCA patient prognosis or exactly how CTSK is involved in the development of THCA.

In the present study, we applied the WGCNA technique to systematically identify genes integral to the EMT and to formulate related coexpression networks. Patients within the TCGA-THCA dataset were stratified based on their EMT scores, yielding two distinct groups—those with high EMT and those with low EMT. Our analysis focused on exploring differences in prognosis and immune cell infiltration between these categorizations. Notably, this study identified CTSK as a critical gene associated with the EMT, emphasizing its substantial role in modulating both tumor proliferation and the metastatic process in THCA. These insights could improve our understanding of the influence of the EMT on THCA pathophysiology and could significantly refine approaches to develop personalized treatment modalities for affected patients.

## 2 Methods

### 2.1 Data sources

Thus, the present study used an analytical dataset consisting of 501 THCA samples derived from the TCGA dataset. This cleaning of the initial data was performed using Perl programming to remove any duplicates or incomplete observations from the dataset. The next procedures were the normalization and annotation of the data to meet the requirements of the subsequent analysis. To determine the genes that were significantly differentially expressed between normal and THCA tissues, the ‘limma’ package within the R environment was used. This analysis used a stringent threshold for statistical analysis where only genes that had a log fold change of ±1 and a P value of less than 0.05 were considered significant. DEG visualization was informative; the chromosomal positions of the DEGs were presented in a circular form using the ‘RCircos’ package. Furthermore, to increase the applicability of this study on the EMT, genes connected to the EMT were selectively incorporated into the analysis. These genes were obtained from the MSigDB and complemented the study with a focused view on biological processes that could underlie the development of THCA and its metastatic spread.

### 2.2 Construction of WGCNA and identification of modules related to the EMT

In this comprehensive analysis, the ‘WGCNA’ package in R was used to identify the gene modules related to the EMT using the TCGA-THCA dataset. The flow of the study began with the identification of the first 1000 genes that showed the highest variability between samples split into two groups with low and high EMT scores. To make the data more suitable for analysis, two suspicious samples, which were determined using cluster analysis, were removed. This was followed by the analysis of scale independence as well as the mean connectivity across the modules at different power levels. This step was necessary for establishing the most appropriate soft threshold that would help in providing a stable analysis of the network with a signed R² value of 0. The 95% confidence interval is considered an adequate level of scale independence. After fixing the soft threshold, the next step was to examine the relationship between the gene expression modules and the EMT parameters. To this end, only those modules that had detectable correlations with the EMT were chosen for further assessment at the level of individual GO terms within the modules. This approach was proposed to uncover not only genes that are highly relevant to the EMT but also genes that are strongly associated with the selected modules based on eigengenes. The GS for each gene in these crucial modules was defined as the absolute value of the correlation with the clinical phenotypes. Moreover, MM was established by plotting the correlation of each gene’s expression pattern to the module eigengene. To depict these findings, scatter plots were created to show the relationship between GS and MM for the genes in the highlighted modules and highlight the complexity of the relationship between these genes and their potential for clinical application.

In our WGCNA analysis, we meticulously selected genes for inclusion in the network based on a series of rigorous criteria aimed at enhancing the validity and interpretability of our findings. Initially, we filtered out genes with low expression levels by setting a threshold where only those genes exhibiting a mean expression value greater than 1 Transcripts Per Million (TPM) across all samples were retained. This step ensured that we focused on genes with sufficient expression for meaningful analysis. Subsequently, we calculated the coefficient of variation (CV) for each gene, which is defined as the ratio of the standard deviation to the mean expression. We included only the top 50% of genes exhibiting the highest CV values, thereby prioritizing those genes that demonstrated significant variability in expression across samples, indicative of their potential biological relevance. Furthermore, to align our analysis with existing biological knowledge, we cross-referenced our gene list with cancer-related genes obtained from well-established databases such as The Cancer Genome Atlas (TCGA) and GeneCards. This additional filtering step allowed us to focus specifically on genes that have documented associations with cancer pathways and processes. After these selection steps, the remaining genes were subjected to the standard WGCNA procedures to construct the co-expression network, wherein we employed a soft-thresholding power to achieve scale-free topology, followed by hierarchical clustering to identify modules of co-expressed genes. This comprehensive approach facilitated the identification of biologically relevant gene modules that may contribute to cancer pathology.

### 2.3 Enrichment analysis of key DEGs

Enrichment analysis of the 68 selected DEGs was performed using the R program’s clusterProfiler package (20). This systematic review included assessments based on both GO and KEGG analyses. To ensure rigorous statistical evaluation, the study adhered to stringent criteria. Therefore, the significance levels were set at an adjusted P value and adjusted q value of less than 0.05. Genes or pathways for which the p value was less than 0.05 were considered to be significantly enriched; this defined the biological relevance of the gene or pathway. In this analysis, the FDR level was set at 0.05 or less to ensure the credibility of the identified gene relationships and pathway impacts.

### 2.4 Construction and validation of the EMT signature

The DEGs were then analyzed using univariate Cox regression analysis with the “tinyarray” package, and LASSO regression, which is a machine learning method, was used. The proposed approach facilitated improved evaluation of the probability effects of specific genes. Of the 68 DEGs, five (CTSK, C3ORF80, FBLN2, PRELP, and SRPX2) had clinical prognostic significance and were included in the prognostic model. In selecting CTSK, C3ORF80, FBLN2, PRELP, and SRPX2 for our EMT signature model, we based the decision on both their statistical significance and biological relevance to EMT and THCA. Each of these genes was identified through a rigorous screening process using WGCNA and LASSO regression, followed by functional enrichment analysis. These five genes stood out due to their significant association with poor patient prognosis and strong involvement in key EMT-related pathways. CTSK was selected because of its well-established role in extracellular matrix degradation, a critical component of EMT. C3ORF80 is involved in cellular processes that contribute to immune regulation and cancer progression. Its expression was correlated with immune infiltration, particularly macrophages and T cells, which are crucial to the tumor microenvironment in THCA. This made C3ORF80 a relevant marker for both immune-related and EMT-driven tumor progression. FBLN2 is part of the extracellular matrix, where it plays a role in stabilizing the structural integrity of tissues. Thus, FBLN2 contributes to our understanding of EMT by highlighting extracellular matrix remodeling in THCA. PRELP is involved in cell-matrix interactions and has been associated with the regulation of EMT through matrix reorganization. SRPX2 was chosen due to its role in angiogenesis and tumor cell invasion, two processes integral to EMT. These five genes together form a robust model that captures both the epithelial and mesenchymal aspects of EMT.

Additionally, to categorize the patients into low-risk and high-risk groups, a median risk score was used. This method highlighted the differences in prognosis between these groups. Risk score: CTSK * 0. 286 + C3ORF80 * 0. 478 - FBLN2 * 0. 636 - PRELP * (-0. 166) + SRPX2 * 0. 310. Subsequently, the TCGA-THCA cohort was split into training and validation sets based on a 2:1 ratio and an 8:1 ratio, respectively. The EMT prognostic model was developed using multiple regression analysis of the coefficients of five critical genes. This robust model facilitated the stratification of the TCGA-THCA cohort into two distinct groups, namely, the high-risk group and the low-risk group, depending on the likelihood of disease progression. To determine DEGs between these risk groups, the Wilcoxon rank-sum test was applied, which demonstrated the genetic differences that led to the different prognoses. To compare the discriminative ability of the EMT model for predicting the 1-, 2-, and 3-year PFIs, ROC curves were constructed. The AUC was calculated using the ‘survivalROC’ package to determine the efficiency of the model in predicting patient prognosis. Furthermore, a Kaplan–Meier estimator was used to compare PFIs among the various risk categories of patients. Statistical analysis of the differences in survival rates was performed using the log rank test at a significance level of p < 0.05, hence validating the model’s ability to identify patients with higher and lower risks of disease progression.

### 2.5 Genomic mutation analysis

The step-by-step approach for obtaining CNV data for the THCA cohort was performed using the R-based ‘TCGAbiolinks’ package through which the GDC portal was accessed. All these analyses were performed using the Genome Reference Consortium Human Build 38 (GRCh38) to avoid variation in genomic alignment. CNV analysis was subsequently performed using the advanced GISTIC2.0 algorithm hosted on the GenePattern platform (21). Genetic analysis was performed on the website http://cloud.genepattern.org/gp/pages/index.jsf using default parameters, including a confidence level of 0.9 to provide statistically accurate results. To display the CNV data that were obtained in the study, the ‘Maftools’ package (22) in R was used to generate a clear map of genomic alterations within the patient population. Furthermore, to better visualize the distribution of highly mutated genes among the clinical subtypes within the THCA samples, waterfall plots were created. These plots were created with the most current version of ‘maftools’ (version 2.12). This process allowed for the compilation and depiction of precise mutation information alongside clinical categorizations, thus improving the understanding of the genomic environment in patients with this type of cancer.

### 2.6 Analysis of immune infiltration

We used three computational algorithms, namely, ssGSEA, xCell, and MCPcounter, to calculate immune infiltration scores, which were visualized using boxplots, stacked plots, correlation scatter plots, and heatmaps. xCell (https://xcell.ucsf.edu/) was used to quantify the infiltration abundance of 67 immune cell types based on transcriptomic data. xCell employs advanced machine learning techniques to derive gene signatures from thousands of diverse cell types, significantly reducing correlations among similar cell types. This approach has been validated using detailed computer simulations that analyze both features and cellular immunophenotyping, demonstrating the effectiveness of xCell in precisely delineating cellular heterogeneity across tissue expression profiles. Next, the ssGSEA method was applied to compute enrichment scores for individual samples and pairs of gene sets, enabling the assessment of the extent of immune infiltration within these samples. Furthermore, the MCPcounter tool was utilized to measure the presence of ten different immune cells within the transcriptomic data, providing a quantitative analysis of immune cell abundance.

### 2.7 Prognostic analysis using CTSK

Using transcriptome data from 513 patients with THCA obtained from the TCGA database, patients were categorized into groups based on high or low CTSK expression, with an established optimal threshold of 3.7326 for gene expression levels. Kaplan–Meier survival curves were then constructed to depict the survival outcomes for both the high-expression and low-expression groups, enabling a comparative analysis of their survival durations.

### 2.8 Cell culture and cell transfection

In this study, the THCA cancer cell lines CAL-62 and KTC-1 were acquired from the American Type Culture Collection (ATCC) and cultured under controlled conditions at 37°C in an atmosphere containing 5% CO_2_. This study employed negative control(NC) and siRNAs specifically targeting CTSK, which were produced by the Tsingke Company (Beijing, China), and the sequences of siRNAs were as follows: siNC(5’-UUCUCCGAACGUGUCACGUTT-3’); siCTSK-1(5’-CAGCAAAGGTGTGTATTATGATGAA-3’); and siCTSK-2(5’-GGUUCAGAAGAUGACUGGA(dT)(dT)-3’). For gene silencing experiments, cells were transiently transfected with either the negative control or CTSK-targeted siRNAs utilizing Lipofectamine 2000 reagent following the protocols provided by Invitrogen.

### 2.9 Quantitative real-time PCR

RNA was isolated from THCA cells with TRIzol reagent (AC0101-B; SparkJade, China). Subsequently, 1 μg of extracted RNA was converted to cDNA utilizing a High-Capacity cDNA Reverse Transcription Kit (Vazyme, R223-01). This cDNA served as the template for subsequent exponential amplification, which was performed using 2 × HQ SYBR qPCR Mix (ZF501; ZOMANBIO; Beijing, China). ACTB served as the internal control for normalization. The forward sequence and reverse sequence of the primers for CTSK were 5’-ACACCCACTGGGAGCTATG-3’ and 5’-GACAGGGGTACTTTGAGTCCA-3’, respectively, and the forward sequence and reverse sequence of the primers for ACTB were 5’-CATGTACGTTGCTATCCAGGC-3’ and 5’-CTCCTTAATGTCACGCACGAT-3’, respectively.

### 2.10 Western blotting

For protein analysis, the collected cell samples were disrupted using RIPA lysis buffer (Catalog No. R0020; Solarbio, Shanghai, China), ensuring thorough cellular breakdown for protein extraction. After cell lysis, protein concentrations were accurately determined with a BCA protein assay kit, allowing for the quantification necessary for further analysis. The proteins were then resolved on SDS−PAGE gels to achieve separation based on molecular weight. After electrophoretic separation, the proteins were carefully transferred onto PVDF membranes obtained from Millipore. The membranes were then incubated with a 5% solution of nonfat milk from Solarbio to block nonspecific binding sites. Primary antibodies directed against CTSK(rabbit polyclonal, 1:1000, A1782, ABclonal), GAPDH(rabbit polyclonal, 1:4000, A19056, ABclonal), N-cadherin (rabbit polyclonal, 1:1000, A21308, ABclonal), vimentin(rabbit polyclonal, 1:4000, A19607, ABclonal), slug(rabbit polyclonal, 1:1000, 9585T, CST) and snail(rabbit polyclonal, 1:1000, 3879T, CST) were applied to the membranes, which were then incubated overnight at a steady temperature of 4°C. Following primary antibody binding, the membranes were exposed to appropriate secondary antibodies, and the appropriate settings were established for detection. The detection phase employed the chemiluminescent method using the Western blotting Detection Kit (ECL; Catalog No. ED0015-A, Sparkjade), ensuring sensitive visualization of the protein bands.

### 2.11 Cell proliferation assay

After transfection, the cells were allowed to adapt for 48 hours before cell activities were assessed. Assessment was conducted using the CCK-8 Cell Proliferation Assay Kit (catalog no. C6005M; US Everbright; Silicon Valley, CA, USA), which strictly adhered to the manufacturer’s instructions. Simultaneously, to evaluate the proliferative responses, the EdU Cell Proliferation Assay Kit (Catalog No. C6015M; US Everbright) was used, which provides a parallel quantitative measure of cell division and growth. For the colony formation assays, an initial seeding density of 1000 cells per well was maintained in six-well plates, and the cultures were incubated for a period ranging between one and two weeks to allow for sufficient colony development. At the conclusion of the incubation period, the colonies were fixed in 4% paraformaldehyde solution for 20 minutes to ensure optimal preservation. Then, colonies were stained with a 0.5% crystal violet solution for 20 minutes to enhance visual contrast for subsequent analysis.

### 2.12 Transwell assay

This study employed Transwell migration assays using 24-well plates with polycarbonate membranes that had an 8-µm pore size (Corning, USA). In these experiments, we filled each lower chamber with 500 µl of RPMI 1640 medium enriched with 10% fetal bovine serum to facilitate cellular growth and migration. In parallel, 200 µl of a serum-free cell suspension, prepared at a density of 1 × 10^6^ cells/ml, was gently pipetted into the upper chamber of the setup. This configuration was maintained in an incubator set at the optimal growth conditions of 37°C and an atmosphere containing 5% CO₂ for a 24-hour period to allow for effective cell migration. After incubation, the cells within the Transwell chambers were fixed in a 5% glutaraldehyde solution to preserve their structure and morphology. Staining was then performed using 0.1% crystal violet dye, allowing the visualization and subsequent analysis of cell migration patterns.

### 2.13 Wound healing

In the described experiment, six-well plates were seeded at a density of 1 × 10^6^ cells per well. Following an overnight incubation period, a deliberate wound was introduced into the confluent cell monolayer utilizing the tip of a 10-µl pipette. Subsequently, the induced scratch was visualized using a high-resolution microscope equipped with options for 10× magnification.

### 2.14 Statistical analysis

Statistical evaluations in this study were conducted utilizing R software, version 4.1.1. Data analysis was performed by using GraphPad Prism 9.0 (San Diego, CA, USA). The two-sided Student’s t-test was used to compare unpaired data. The Cox hazard regression model was used for univariate analysis, P value < 0.05 was considered statistically significant.

The analyses included one-way and multifactorial Cox regression using ‘survival’ and ‘survminer’ packages available within R, respectively. The criterion for statistical significance was set such that a p value less than 0.05 indicated statistical significance.

## 3 Results

### 3.1 Construction of the coexpression network in THCA

**Figure 1:**
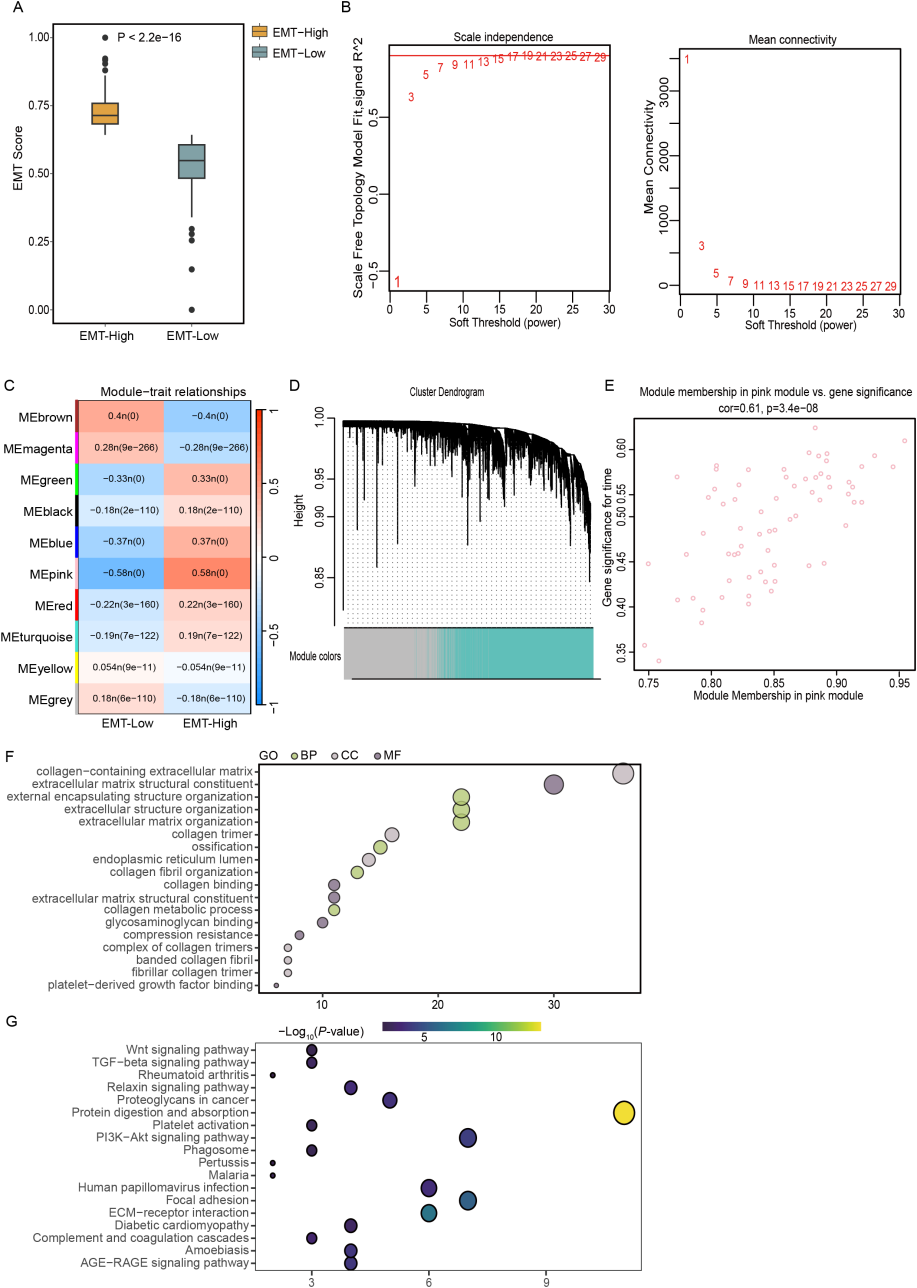
Elucidating EMT dynamics and key genetic players in THCA using TCGA data. **(A)** Classification of TCGA-THCA samples into EMT-High and EMT-Low groups using the ssGSEA algorithm. **(B)** Determination of the optimal soft-thresholding power at 3, illustrated using graphs depicting scale independence and mean connectivity for assessing scale-free network topology. **(C)** Correlation analysis between gene modules and EMT scores to identify relevant genetic interactions. **(D)** Construction of a coexpression network using WGCNA based on RNA-seq profiles from the TCGA-THCA dataset. **(E)** Scatter plot highlighting the pink module, where key genes with a GS greater than 0.5 and MM above 0.7 were identified, indicating significant topological overlap. **(F, G)** Functional enrichment analyses using GO and KEGG pathway analyses to explore the biological implications of genes within the EMT-based signature.

In this detailed study, we harnessed the EMT gene set from the MSigDB website to conduct gene pathway assessments for 501 patients diagnosed with THCA using the ssGSEA algorithm. Patients were divided into two groups according to a median EMT score of 0.67. The group with scores above this median, termed the EMT-High group, demonstrated significantly elevated EMT scores that were greater than those in the EMT-Low group, with statistical analyses confirming a significant difference (p < 2.2e-16), as detailed in **Figure 1A**. Furthermore, to explore the gene expression profiles across these patients, WGCNA was employed to scrutinize the expression data of 14,564 genes collected from the 501 THCA samples. Through meticulous determination, a soft-thresholding power of 15 was established based on achieving a scale-free topology criterion with an R² value of 0.9, as depicted in **Figure 1B**. The analytical process led to the identification of ten distinct gene modules after setting the dissolution threshold (DissThres) to 0.2 to merge dynamic modules. Notably, cluster dendrogram analysis revealed that the pink module had the most substantial correlation with the EMT scores, with a Pearson correlation coefficient of 0.58 and a statistically significant p value of 0, as illustrated in **Figure 1C**. Given the focus of our research on the EMT phenomenon within the TCGA-THCA dataset, the green module was identified as a hub module. This module’s pivotal role is highlighted in **Figure 1D**, underscoring its relevance in our ongoing analysis. To further refine our study, thresholds for GS and MM were set at greater than 0.5 and 0.7, respectively. This stringent criterion facilitated the identification and selection of 68 key genes that exhibited strong associations with EMT characteristics, paving the way for subsequent detailed investigations. These pivotal genes are shown in **Figure 1E**, setting the stage for future exploratory and confirmatory studies.

### 3.2 Functional analyses of EMT-related genes

The functional enrichment analysis conducted in this study revealed a significant concentration of GO terms associated with components of the extracellular matrix. Notably, these included terms related to collagen-enriched extracellular matrices, the structural constituents of such matrices, and the organization of encapsulating structures external to cells. The analysis also highlighted significant enrichment in terms related to the broader organization of extracellular structures and matrices themselves (**Figure 1F**). KEGG pathway analysis revealed the enrichment of specific pathways that play pivotal roles in cellular interactions and signaling mechanisms. The identified pathways included the PI3K-Akt signaling pathway, protein digestion and absorption, ECM-receptor interactions, and focal adhesion, which are all essential for cellular communication and adhesion processes (**Figure 1G**). The enrichment of these pathways suggested that genes associated with EMT may actively contribute to the malignant progression of THCA by enhancing the activation of these critical signaling pathways. This activation potentially facilitates the invasive and metastatic behavior of cancer cells, underlining the importance of these pathways in the context of cancer progression and the potential for targeted therapeutic interventions.

### 3.3 Construction of the EMT-based signature

**Figure 2:**
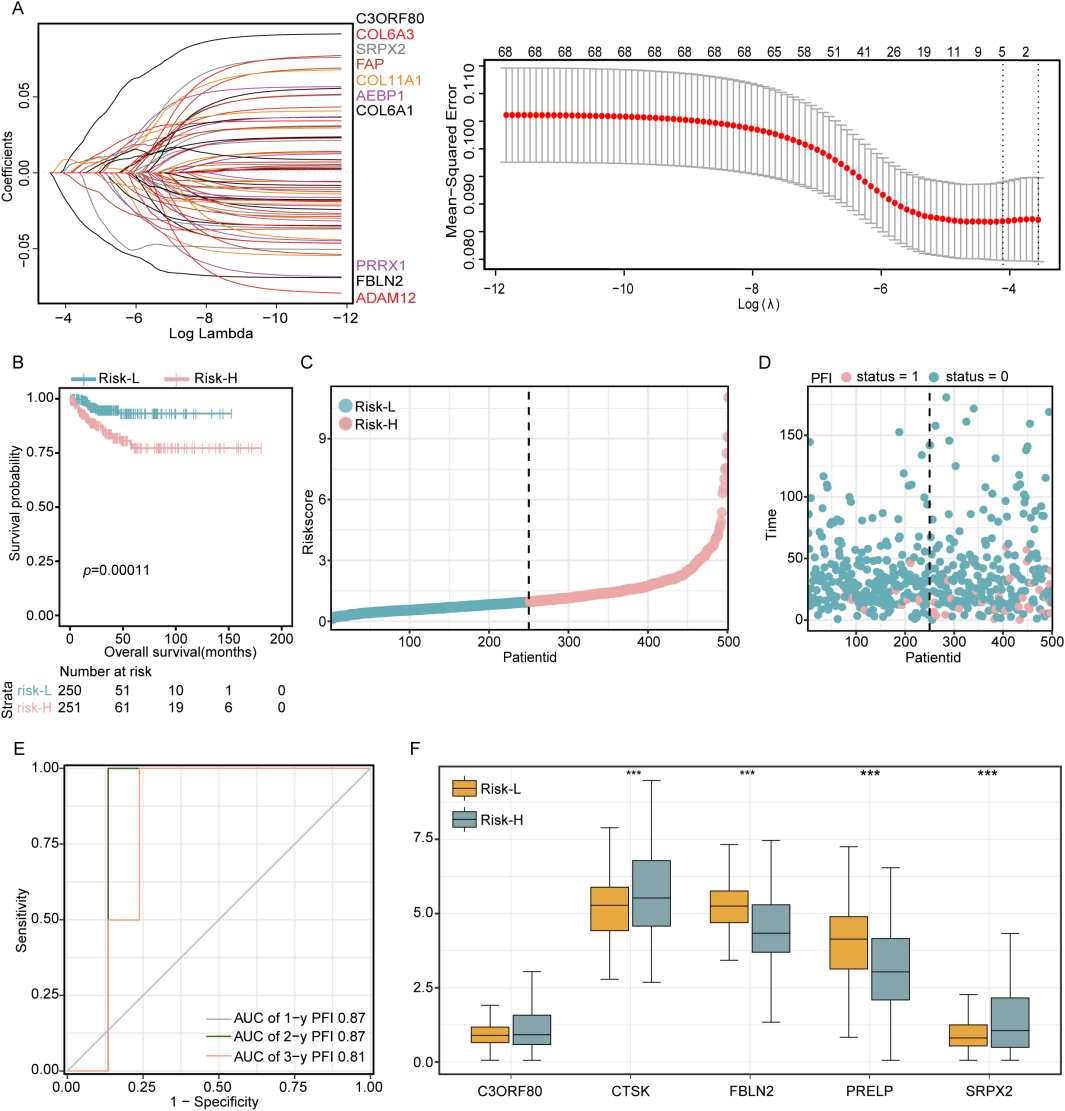
Development of a 68-gene prognostic signature based on differential expression analysis in two subtypes. **(A)** LASSO regression was applied to refine the gene selection for the prognostic model. **(B)** Kaplan−Meier survival curves delineating the outcomes of 509 patients stratified into high-risk and low-risk groups according to their EMT scores. **(C, D)** Presentation of risk curves illustrating the distribution of prognostic scores along with patient survival time and status. **(E)** Time-dependent ROC curves evaluating the predictive accuracy of survival probabilities based on DEGs. **(F)** Comparative analysis of the expression levels of five critical DEGs between patients in the low-risk and high-risk groups. Significance levels are denoted as ***P<0.001.

In this analysis, a LASSO regression approach was utilized to scrutinize the prognostic potential of 68 genes, and a critical minimum value of 5 was determined (**Figure 2A**). This analysis identified five genes with significant characteristics related to EMT: C3ORF80, CTSK, FBLN2, PRELP, and SRPX2. These genes were then used to construct a robust EMT risk score model. The model was formulated as follows: EMT risk score = (CTSK * 0.286) + (C3ORF80 * 0.478) + (FBLN2 * -0.636) + (PRELP * -0.166) + (SRPX2 * 0.310). Using this predictive model, patients with THCA were stratified into two distinct risk categories based on the median risk score of the cohort. The categorization placed 84 patients in the high-risk group, which corresponded with a markedly increased mortality rate. In contrast, the classification identified 83 patients as belonging to the low-risk group, which was associated with significantly enhanced survival rates, as depicted in **Figures 2C, D**. The disparity in survival probabilities between these groups was starkly illustrated in the Kaplan−Meier survival plots (**Figure 2B**), indicating a significantly shorter survival duration for patients in the high-risk group than for those in the low-risk group. Furthermore, the reliability of the EMT risk score was evaluated using receiver operating characteristic (ROC) curve analysis, yielding areas under the curve (AUCs) for 1-year, 2-year, and 3-year survival predictions of 0.87, 0.87, and 0.81, respectively, for the TCGA-THCA cohort (**Figure 2E**). This analysis underscores the prognostic accuracy of the EMT risk score model in predicting patient outcomes. Additionally, a comparative analysis of gene expression within these risk groups revealed that CTSK and SRPX2 were expressed at higher levels in the high-risk group, whereas FBLN2 and PRELP showed reduced expression levels in the same group compared to the low-risk group (**Figure 2F**). This differential expression pattern further corroborates the link between these genes and the aggressive clinical behavior associated with higher EMT risk scores.

### 3.4 Validation of the training set and validation set

**Figure 3:**
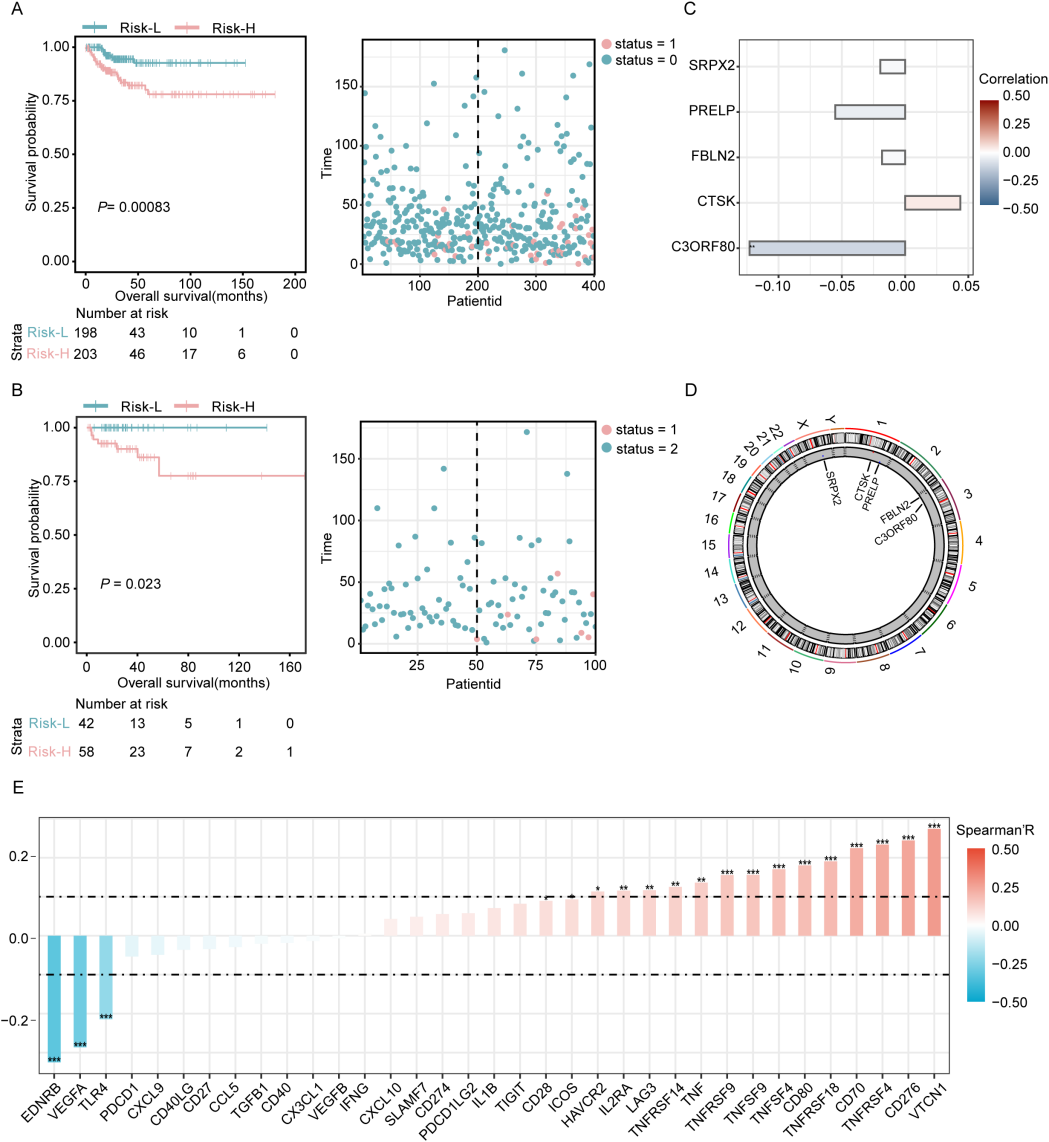
Prognostic evaluation in training and validation sets with examination of key genes. **(A)** Visualization of the risk curves showing the distribution of prognostic scores and survival statuses within the training cohort. **(B)** Risk curves depicting the prognostic scores and survival statuses across the validation cohort. **(C)** Analysis of the correlation between key genes and patient prognosis. **(D)** A circular chromosome plot illustrating the genomic positions of key genes relevant to the study. **(E)** Evaluation of the associations between the risk score model and 43 immune checkpoint genes conducted using Spearman’s correlation coefficient. Significance levels are denoted as *P<0.05, **P<0.01, and ***P<0.001.

The dataset was divided into two comprehensive sections: approximately 80% were assigned to the training set, and the remaining 20% formed the validation set. Subsequent analyses of the training data indicated that the prognosis for patients identified as belonging to the high-risk group was significantly less favorable than that for those assigned to the low-risk group. This disparity was particularly evident in the elevated mortality rates observed among individuals in the high-risk category (as detailed in **Figure 3A**). Similarly, evaluation of the validation set demonstrated consistent results with both the training set and the entire dataset, confirming the reproducibility and robustness of the findings across different subsets of data (**Figure 3B**). Based on the previously described LASSO linear regression, after removing redundant genes and constructing a risk model, we ultimately screened five DEGs (SRPX2, PRELP, FBLN2, CTSK, and C3ORF80). Of these, C3ORF80 expression showed a significant positive correlation with prognosis, whereas the expression of the other four genes exhibited no significant correlation with prognosis (**Figure 3C**). In addition, the chromosome circle plot illustrated the chromosomal locations of SRPX2, PRELP, FBLN2, CTSK and C3ORF80 (**Figure 3D**). Additionally, Spearman correlation analysis revealed significant negative correlations between the risk score and EDRNB and VEGFA, whereas positive correlations were observed with VTCN1, CD276, and TNFRSF4 (**Figure 3E**).

### 3.5 Construction of the nomogram and mutation analysis

**Figure 4:**
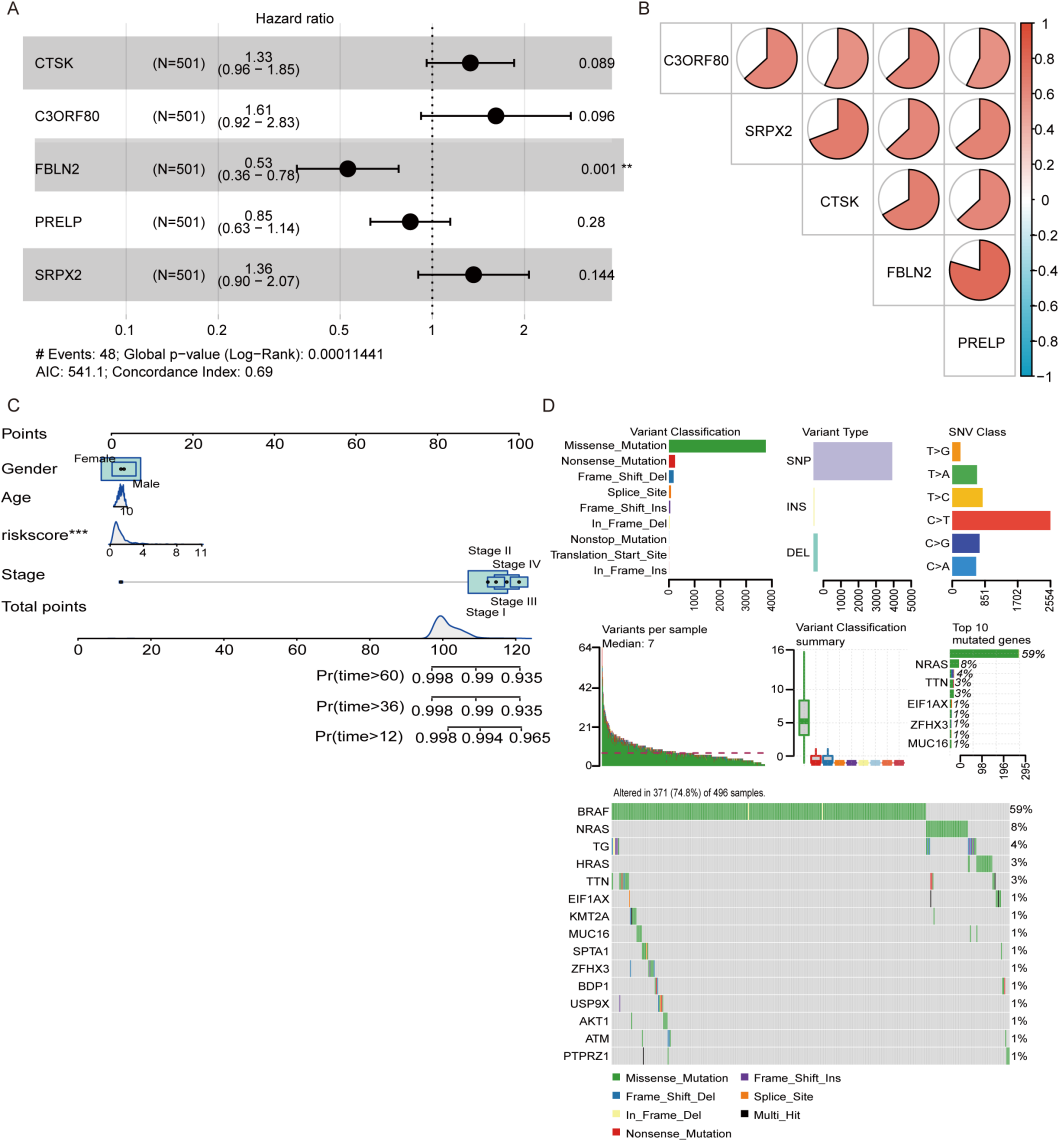
Development of a nomogram and analysis of genetic mutations. **(A)** Multivariate analysis was conducted to confirm the independent prognostic factors influencing patient outcomes. **(B)** Pie chart illustrating the interrelationships among the genes included in the model. **(C)** A nomogram was constructed that incorporates sex, age, risk score, and cancer stage to predict the risk of recurrence at 12, 36, and 60 months. **(D)** Waterfall plot displaying the spectrum of single nucleotide variant (SNV) mutations in the genes modeled, highlighting the genetic alterations within the study cohort. **P<0.01, ***P<0.001.

Among the five DEGs screened, only FBLN2 exhibited a significant association with the hazard ratio (p=0.001), which was lower in patients in whom FBLN2 was highly expressed (**Figure 4A**). The interrelationships among these 5 genes are depicted in **Figure 4B**, where FBLN2 displayed the strongest correlation with PRELP. To evaluate the recurrence risk of individual THCA patients, a nomogram incorporating four predictive factors, namely, gender, age, risk score, and cancer stage, was developed. This tool indicates that for THCA patients with a high genetic risk score (GRS) and N0 stage disease, the probabilities of recurrence at 12, 36, and 60 months are estimated to be 0.127, 0.403, and 0.426, respectively (**Figure 4C**). Additionally, analysis of single nucleotide variants revealed that missense mutations were the predominant type of DNA mutation found within the five DEGs. Among these, single nucleotide polymorphisms (SNPs) are the most frequently occurring mutations, with transitions from cytosine to thymine representing the most common type of base substitution observed. In addition, BRAF, NRAS and HRAS were the most commonly mutated genes in THCA, and most of their mutations were missense mutations (**Figure 4D**).

### 3.6 Evaluation of the immune microenvironment

**Figure 5:**
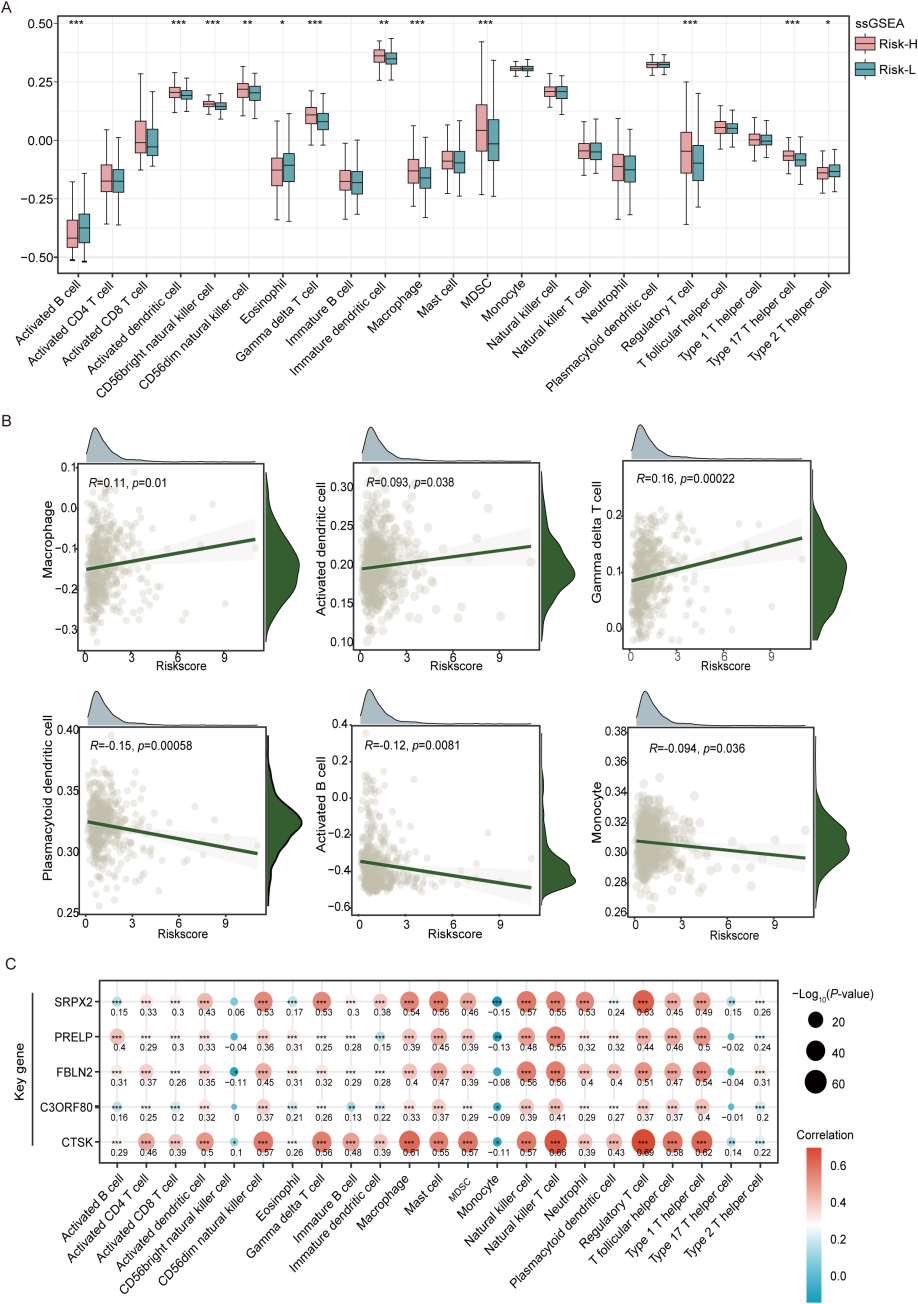
Analysis of tumor immune microenvironment variations in the high-risk and low-risk groups of the TCGA-THCA cohort. **(A)** Box plots reveal the variation in the levels of 23 different immune cell types between groups classified as high- and low-risk, as established by ssGSEA. **(B)** A scatterplot illustrates the correlation between the risk score and the distribution of different immune cell types within the tumor microenvironment. **(C)** The heatmap visualizes correlation coefficients linking crucial genes with immune cells, where red dots represent positive correlations, blue dots signify negative correlations, and the star symbol (*) highlights statistically significant findings. *P<0.05, **P<0.01, ***P<0.001.

Using the ssGSEA algorithm to analyze the composition of tumor-infiltrating immune cells, we observed distinct profiles in different risk groups of tumor patients. This study revealed increased levels of various immune cells, including CD56dim natural killer cells, gamma delta T cells, CD56bright natural killer cells, immature dendritic cells, macrophages, MDSCs, activated dendritic cells, regulatory T cells, and type 17 T helper cells, in the high-risk group, all of which demonstrated statistically significant differences (p < 0.05). Conversely, the low-risk group exhibited significantly greater numbers of activated B cells, eosinophils, and type 2 T helper cells (p < 0.05), as shown in **Figure 5A**. Higher levels of macrophage infiltration, MDSC infiltration, and regulatory T-cells (Tregs) in the high-risk group suggest that the high-risk group may have more significant signs of immune evasion. The correlation scatter plot illustrates that the EMT risk score is positively associated with the infiltration of certain immune cells, including macrophages, activated dendritic cells, and gamma delta T cells. Conversely, this score showed a negative correlation with plasmacytoid dendritic cells, activated B cells, and monocytes, as depicted in **Figure 5B**. This analysis highlights the differential relationships between the EMT risk score and specific immune cell types, suggesting varying influences of these cells on EMT progression. In this analysis, we also selected 23 immune cells expressed in the TCGA cohort for analysis and calculated the correlation coefficients between the expression levels of the five genes and their infiltration levels. The results of the thermographic analysis are shown in **Figure 5C**. Among them, CTSK showed a significant positive correlation with regulatory T cells, macrophages, type I T helper cells, and natural killer cell (all R>0.6 and all p<0.001). SRPX2 also showed a positive correlation with regulatory T cells (R>0.6 and all p<0.001).

**Figure 6:**
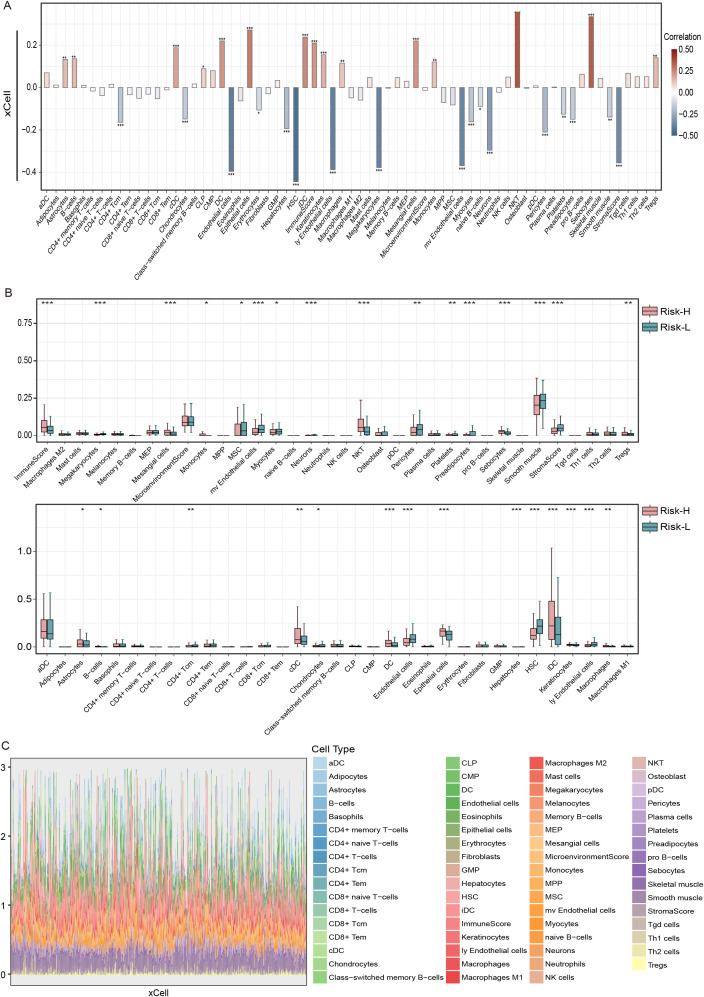
The correlation between risk score and immune cells types in THCA using the xCell algorithm and the TCGA dataset. **(A)** Bar graph of the risk score based on the xCell immune infiltration algorithm, **(B)** correlation boxplots of the risk score and 23 xCell immune cells, and **(C)** immune cell stacking plots of xCells from 501 thyroid cancer patients. ns = non-significant, *P<0.05, **P<0.01, ***P<0.001.

Using the xCell algorithm, our research investigated the correlation between tumor-infiltrating immune cells and the EMT risk score in THCA patients. This study revealed a robust positive correlation between the risk score and NK T cells, with a correlation coefficient exceeding 0.3 and a p value less than 0.001, confirming statistical significance (**Figure 6A**). Conversely, the most substantial negative correlation was observed with HSCs, where the correlation coefficient was less than -0.4, and the p value was less than 0.001.

Moreover, boxplot analyses highlighted that immune infiltration levels varied significantly between risk groups. Individuals categorized within the low-risk group displayed elevated levels of MSCs, microvascular endothelial cells, myocytes, and HSCs relative to their counterparts in the high-risk group. Conversely, the high-risk group was characterized by increased quantities of monocytes, NK T cells, sebocytes, Tregs, immature dendritic cells (iDCs), and macrophages. Additionally, high-risk patients had a greater overall immune score, whereas low-risk patients had an elevated stromal score, indicating a differential stromal contribution to the tumor microenvironment (**Figure 6B**). The stacked diagrams provided a detailed view of the immune cell infiltration landscape across individual patients, revealing notable differences in the proportions of infiltrating immune cell subsets among them (**Figure 6C**).

**Figure 7:**
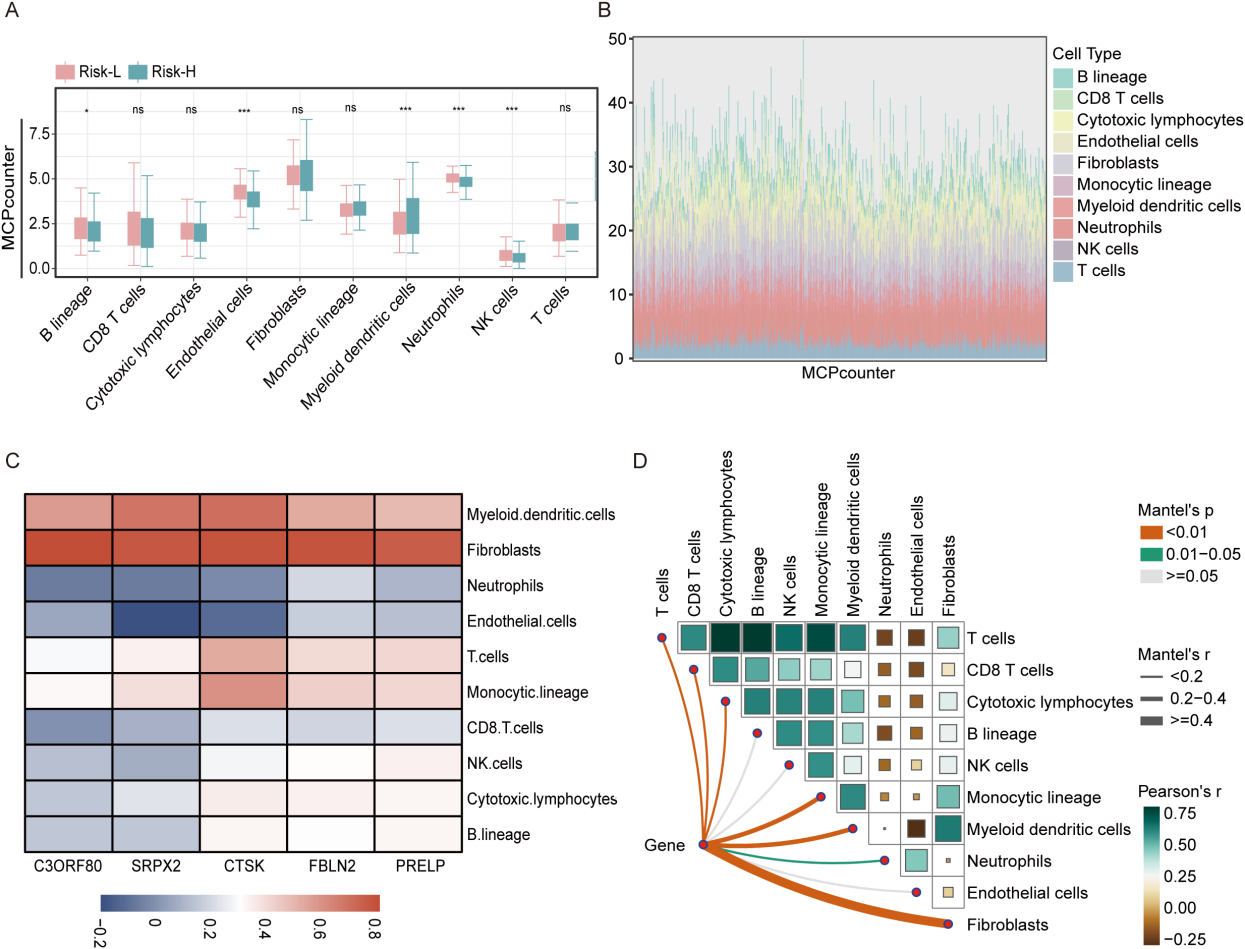
Analysis of the association between the risk score and immune cell types in the THCA using the MCPcounter approach. **(A)** A boxplot illustrates the variation in immune cell infiltration among the high-risk and low-risk categories as determined using the MCPcounter algorithm within the TCGA dataset. **(B)** Stacked bar chart illustrating the distribution of immune cells across 501 thyroid cancer patients as analyzed using MCPcounter. **(C)** Heatmap displaying the correlations between five key genes and the levels of various immune cell types, as quantified using MCPcounter. **(D)** LINKET map showing the relationships between the abundances of immune cells linked to ten specific immune cell genes and model genes within the immune microenvironment. ns = non-significant, *P<0.05, ***P<0.001.

In the extended analysis of the THCA, we applied the MCPcounter algorithm to determine the associations between the concentrations of immune cells infiltrating tumors and risk scores. Detailed boxplot assessments demonstrated significantly greater numbers of B lineage cells, endothelial cells, neutrophils, and NK cells in the high-risk group than in the low-risk group. These findings suggest a pronounced immunological signature that correlates with an increased risk of tumor development (**Figure 7A**). Additionally, using stacked diagrams, we observed that the proportions of infiltrated immune cell subsets varied significantly across patients, highlighting the diverse immune landscape present within the patient cohort (**Figure 7B**). The heatmap showed that all five EMT-related genes were positively associated with fibroblasts, whereas SRPX2 and CTSK were negatively associated with endothelial cells and neutrophils, as shown in **Figures 7C, D**.

### 3.7 CTSK potentially play an important oncogenic role in THCA

**Figure 8:**
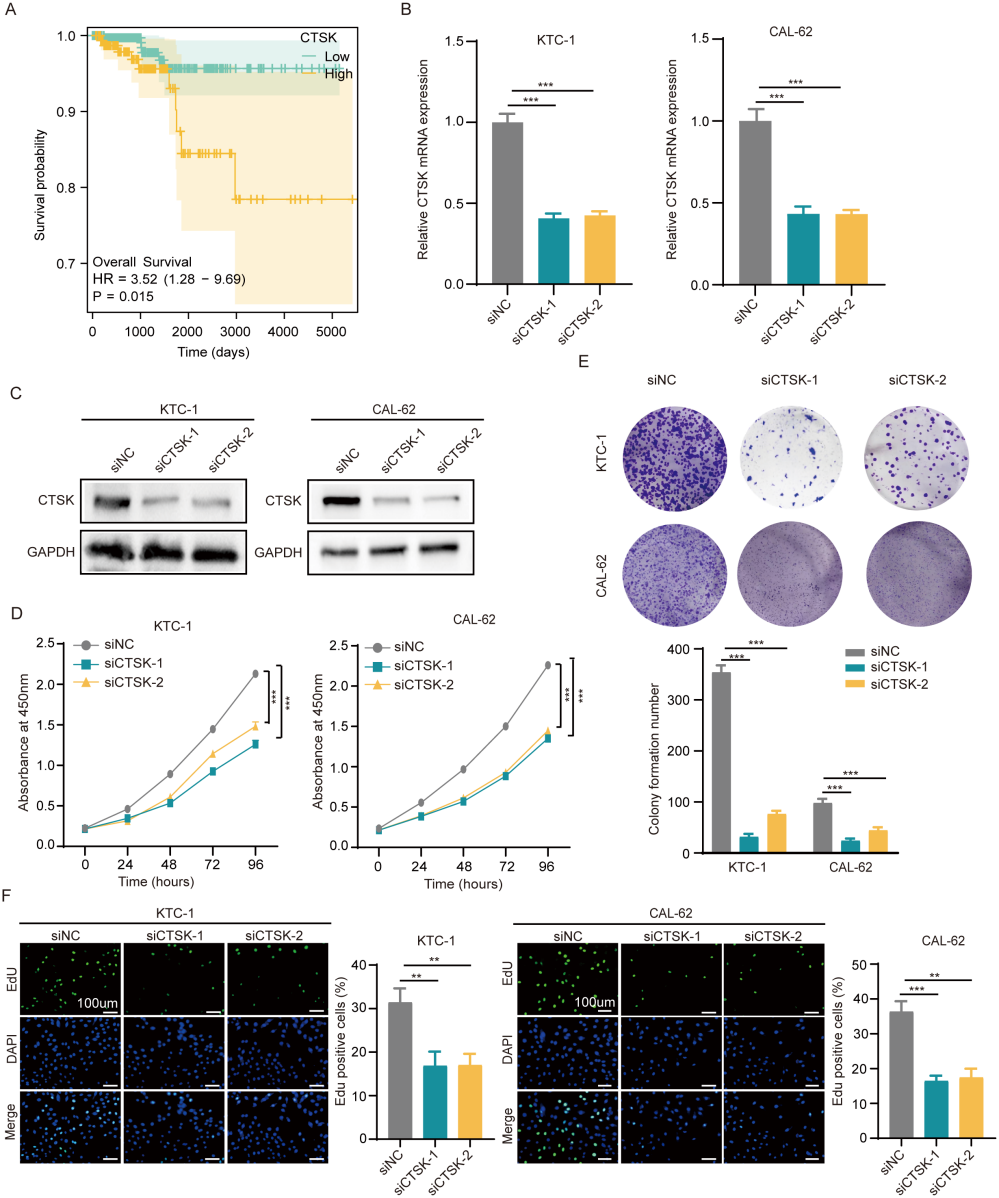
CTSK knockdown inhibits cell proliferation and metastasis. **(A)** Correlation between the CTSK expression level and overall survival of THCA patients. **(B, C)** The knockdown efficiency of CTSK at the gene level was verified using RT−qPCR and western blotting. **(D)** CCK-8 assays revealed that KTC-1 and cal-62 cells with CTSK knockdown exhibited significantly weaker cell activity than siNC cells. **(E)** The colony formation assay demonstrated that the colony formation ability of KTC-1 and cal-62 cells in which CTSK was knocked down was substantially lower than that of cells from the siNC control group. **(F)** The results of the EdU incorporation assay showed that the proliferation of CTSK-knockdown KTC-1 and CTSK-knockdown cal-62 cells was significantly lower than that of siNC-transfected cells. **P<0.01, ***P<0.001. Scale bar =100 μm.

Further investigations have been conducted to explore the correlation between CTSK expression levels in overall THCA patient and individual patient outcomes. According to previously published data, the CTSK scores in the group at high risk were much greater than those in the group at low risk (**Figure 2F**). Our recent analysis builds on these findings, demonstrating that increased CTSK expression is significantly correlated with a decrease in patient prognosis (p=0.015, **Figure 8A**).

**Figure 9:**
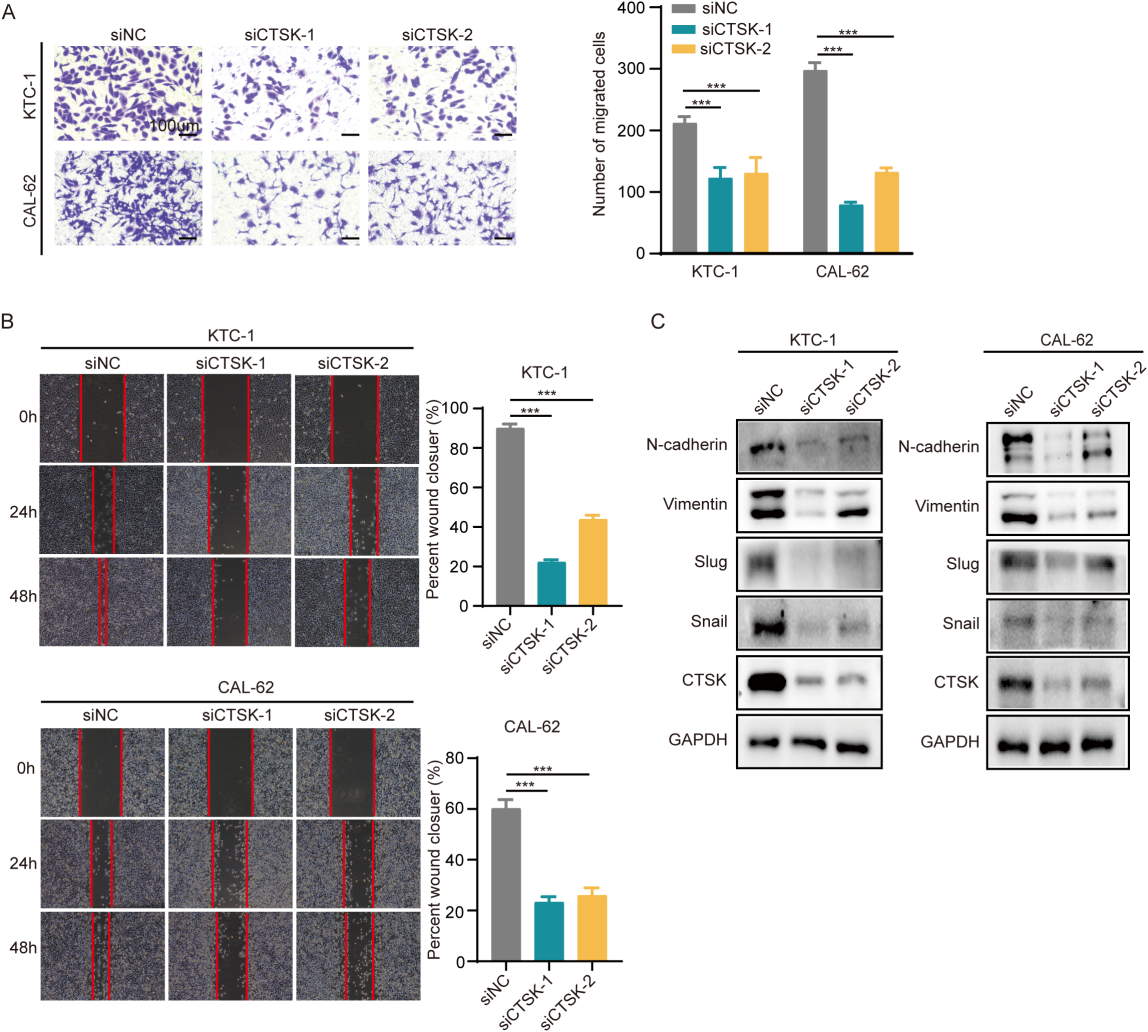
CTSK knockdown inhibits cell motility and migration. **(A)** A Transwell assay revealed reduced cellular mobility in CTSK-knockdown KTC-1 and cal-62 cells compared to control cells, demonstrating a significant reduction in the ability of these cells to traverse membrane pores. **(B)** Wound healing assays at 24 hours postwound creation revealed decreased motility in CTSK-knockdown KTC-1 and CTSK-knockdown cal-62 cells compared with that in the siNC group, as indicated by decreased closure rates. **(C)** Western blot analysis showing decreased levels of EMT markers, including Vimentin, N-cadherin, Snail, and Slug, in KTC-1 and CAL-62 cells following CTSK knockdown, with GAPDH serving as the loading control. The reduction in these proteins underscores significant suppression of EMT progression. ***P<0.001. Scale bar =100 μm.

To investigate the impact of CTSK suppression on cellular dynamics, functional experiments were conducted using the KTC-1 and Cal-62 thyroid carcinoma cell lines. Initially, the effectiveness of CTSK knockdown was validated using RT−qPCR and Western blot analyses, demonstrating a significant reduction in CTSK expression (p < 0.001, **Figures 8B, C**). Subsequently, a series of assays were performed to assess cellular functions after knockdown. After CTSK elimination, the CCK-8 assay, colony formation assay, and EdU assay all revealed significant reductions in cell activity, colony development, and proliferation. This reduction was statistically significant (p < 0.01, p < 0.001; **Figures 8D–F**). Furthermore, Transwell and wound healing experiments demonstrated that cell motility and migration were substantially impaired in response to CTSK knockdown (p < 0.01, p < 0.001; **Figures 9A, B**). Following CTSK knockdown, Western blot analysis revealed a significant decrease in the expression of N-cadherin, Vimentin, Slug, and Snail (**Figure 9C**).

## 4 Discussion

The majority of differentiated thyroid cancers exhibit a favorable prognosis. For these patients, the primary treatment modality is surgery, followed by subsequent radioactive iodine ablation (iodine-131) or thyroxine therapy. However, given that some patients with THCA are prone to tumor metastasis and recurrence or even progression to fatal THCA, systemic treatment is needed, and targeted therapies are preferred (23). The EMT is strongly associated with poor prognosis in THCA patients, and the EMT properties of THCA make therapy targeting EMT-related genes an attractive therapeutic option (24). Remarkably, the potential and functions of EMT-related genes in THCA remain largely unexplored.

In recent years, precision medicine has revolutionized cancer treatment by aiming to personalize disease prevention and treatment strategies through the analysis of individual variations in genomics, the external environment, and lifestyle. An increasing number of researchers have already established subgroups based on the molecular profiles of patients, representing different phenotypes, prognoses and treatment responses. In the context of precision medicine, recent studies have illustrated the importance of gene expression profiling in various cancers. For example, in acute myeloid leukemia (AML), profiling based on the expression of genes linked to ferroptosis can identify a subset of patients with a poorer prognosis who may benefit from ferroptosis-inducing treatments (25). Patients with colorectal cancer (CRC) are stratified into high-risk and low-risk groups using patterns of autophagy-related gene expression, and this information facilitates decision making regarding more aggressive treatments (26). Moreover, in gastric cancer (GC), categorizing patients into subtypes based on RNA n6-methyladenosine-related regulator expression revealed that those patients in certain high-risk subtypes demonstrate significant resistance to immunotherapy (27).

During this investigation, we identified two distinct expression profiles linked to the EMT, designated as the EMT-high and EMT-low categories. These groups exhibited significant differences in terms of prognosis, with the EMT-high group demonstrating a poorer prognosis than the EMT-low group. ssGSEA showed more macrophage infiltration, MDSC infiltration and regulatory T cell (Treg) expression in the EMT-high group compared to the EMT-low group, all three of which modulate the immune response by inhibiting the activity of effector T cells and other immune cells, thereby suppressing the anti-tumor immune response and promoting tumor growth. The xCell algorithm revealed that Treg levels were generally greater in patients in the high-risk subgroup than in those in the low-risk subgroup, indicating a potential association between the EMT-high subgroup and immune evasion through Treg activation. Numerous studies have reported that oncogenes induce malignant progression of tumors by activating both Treg cells and the EMT and that Treg cells can also induce the EMT in tumor cells (27, 28). The findings from this study indicate that Treg cells significantly influence THCA progression.

Based on the DEGs from the two groups, we identified five genes (SRPX2, PRELP, FBLN2, CTSK and C3ORF80) for the construction of prognostic models using one-way and LASSO Cox analyses. The five EMT-related genes identified here offer significant potential for clinical application, particularly in personalized medicine for THCA. These EMT biomarkers can categorize patients into distinct risk groups based on their EMT signatures. Patients with higher EMT scores, associated with elevated CTSK and SRPX2 expression, tend to have a worse prognosis. This stratification provides clinicians with valuable information on disease progression risk, enabling more intensive monitoring for high-risk individuals. For example, such patients could be prioritized for frequent imaging and biomarker assessments to ensure early detection of recurrence or metastasis. Integrating EMT-related biomarkers into clinical practice could significantly enhance precision medicine approaches. By considering the EMT signature in treatment planning, oncologists can customize therapies based on the tumor’s molecular characteristics. Additionally, the EMT signature could identify patients who may be less responsive to conventional treatments like radioactive iodine, guiding them toward alternative therapies.Moreover, these EMT-related genes could be developed into a biomarker panel for early detection and regular screening of THCA patients. Detecting elevated levels of these genes in blood samples or biopsy tissues could help identify patients at higher risk of disease progression or recurrence before clinical symptoms arise. This early detection could improve survival outcomes by enabling prompt interventions.

Research has indicated an association between CTSK expression and the malignant advancement of various tumors. In prostate cancer, molecules downstream of CTSK act as control elements that regulate the expression of EMT-related genes and promote PC cell metastasis and hyperproliferation (19). CTSK has emerged as a crucial mediator linking gut microbiota dysbiosis to CRC metastasis, thereby contributing significantly to the invasive phenotype of CRC cells both *in vitro* and *in vivo* (29). Research on hepatocellular carcinoma (HCC) revealed that CTSK significantly influences cell proliferation. This action is accomplished through its interaction with the SIAH1/protein kinase B (AKT) signaling pathway, where CTSK enhances SIAH1 protein ubiquitination, thereby promoting HCC cell growth and proliferation (17). Although extensive research has been conducted on the biological functions of CTSK in various tumors, limited knowledge exists regarding its involvement in the biological processes of THCA. This study demonstrates that CTSK is linked to poor prognosis in thyroid cancer (THCA) and actively promotes the proliferation and migration of THCA cells. Additionally, it increases the expression of key epithelial-mesenchymal transition (EMT) markers, including N-cadherin, vimentin, slug, and snail, as shown by *in vitro* experiments. CTSK, a critical factor in extracellular matrix degradation and immune modulation, emerges as a promising therapeutic target in THCA. Targeting CTSK, especially in patients with high CTSK expression, may improve outcomes for those in the high-risk EMT group. Additionally, the relationship between EMT signatures and immune cell infiltration, particularly macrophages, myeloid-derived suppressor cells (MDSCs), and regulatory T cells, opens pathways for combining these biomarkers with immunotherapy. High-risk patients with increased immunosuppressive cell infiltration might benefit from treatments that reactivate the immune system, such as immune checkpoint inhibitors. Furthermore, EMT profiles could serve as predictive biomarkers for selecting suitable candidates for immunotherapy. We can also explore whether the combination of CTSK-targeted therapy with immunotherapy or chemotherapy can further improve the therapeutic efficacy of THCA, which can provide a basis for clinical personalized treatment.

Although we obtained the above analyses in this study and some of the results have been validated by *in vitro* experiments, there are still some shortcomings in this study. Firstly, there are inherent limitations of the data in the TCGA database, for example, the number of samples in the TCGA-THCA dataset is relatively small, which may lead to insufficient efficacy of statistical analyses to detect biomarkers or gene variants with small effect sizes. In addition, although the TCGA database provides a wealth of transcriptomic data, these data originate from multiple technology platforms, and technical differences between these platforms may also lead to inconsistencies in the data, as well as increasing the complexity of data integration and data analysis. Finally, we have only validated our analyses by *in vitro* cytological experiments and have not yet completed *in vivo* experiments; in the future, further refinement of the *in vivo* experiments as well as exploring the role of CTSK in immune cell infiltration will be the main focus of our research.

## 5 Conclusions

In conclusion, we identified and validated the key gene CTSK, which is closely related to the EMT in THCA, and we concluded that CTSK could serve as an important biomarker to assist in the diagnosis of THCA.

## Data Availability

The original contributions presented in the study are included in the article/supplementary material. Further inquiries can be directed to the corresponding author.
